# Effects of Particle Size Fractions on Reducing Heart Rate Variability in Cardiac and Hypertensive Patients

**DOI:** 10.1289/ehp.8145

**Published:** 2005-08-08

**Authors:** Kai-Jen Chuang, Chang-Chuan Chan, Nan-Ting Chen, Ta-Chen Su, Lian-Yu Lin

**Affiliations:** 1 Institute of Occupational Medicine and Industrial Hygiene, College of Public Health, National Taiwan University, Taipei, Taiwan; 2 Department of Internal Medicine, National Taiwan University Hospital, Taipei, Taiwan

**Keywords:** air pollution, autonomic system, epidemiology, heart rate variability, particulate matter

## Abstract

It is still unknown whether the associations between particulate matter (PM) and heart rate variability (HRV) differ by particle sizes with aerodynamic diameters between 0.3 μm and 1.0 μm (PM_0.3–1.0_), between 1.0 μm and 2.5 μm (PM_1.0–2.5_), and between 2.5 μm and 10 μm (PM_2.5–10_). We measured electrocardiographics and PM exposures in 10 patients with coronary heart disease and 16 patients with either prehypertension or hypertension. The outcome variables were standard deviation of all normal-to-normal (NN) intervals (SDNN), the square root of the mean of the sum of the squares of differences between adjacent NN intervals (r-MSSD), low frequency (LF; 0.04–0.15 Hz), high frequency (HF; 0.15–0.40 Hz), and LF:HF ratio for HRV. The pollution variables were mass concentrations of PM_0.3–1.0_, PM_1.0–2.5_, and PM_2.5–10_. We used linear mixed-effects models to examine the association between PM exposures and log_10_-transformed HRV indices, adjusting for key personal and environmental attributes. We found that PM_0.3–1.0_ exposures at 1- to 4-hr moving averages were associated with SDNN and r-MSSD in both cardiac and hypertensive patients. For an interquartile increase in PM_0.3–1.0_, there were 1.49–4.88% decreases in SDNN and 2.73–8.25% decreases in r-MSSD. PM_0.3–1.0_ exposures were also associated with decreases in LF and HF for hypertensive patients at 1- to 3-hr moving averages except for cardiac patients at moving averages of 2 or 3 hr. By contrast, we found that HRV was not associated with either PM_1.0–2.5_ or PM_2.5–10_. HRV reduction in susceptible population was associated with PM_0.3–1.0_ but was not associated with either PM_1.0–2.5_ or PM_2.5–10_.

The association between particulate air pollution and cardiovascular mortality rate and hospitalization has been reported in epidemiologic studies (Koken et al. 2003; Pope et al. 1999, 2004a). In most epidemiologic studies, particulate matter (PM) has been characterized as the mass concentration of coarse particles with aerodynamic diameters < 10 μm (PM_10_) and fine particles with aerodynamic diameters < 2.5 μm (PM_2.5_). The association appears to be more evident as particle size gets smaller. Schwartz et al. (1996) reported that the association between PM and daily mortality rates was more evident with exposure to PM_2.5_ than to PM_10_. By examining the relationship between air pollution and cardiopulmonary health in elderly subjects with coronary heart disease (CHD), de Hartog et al. (2003) showed that PM_2.5_ had a greater association with some cardiac symptoms than did PM_10_. Several panel studies also demonstrated that decreased heart rate variability (HRV) was separately associated with either mass concentrations of PM_10_ (Gold et al. 2000; Pope et al. 1999) and PM_2.5_ (Creason et al. 2001; Gold et al. 2000; Holguin et al. 2003; Liao et al. 1999; Magari et al. 2001, 2002; Park et al. 2005; Pope et al. 2004b; Riediker et al. 2004) or number concentrations of submicrometer particles with a size range of 0.02–1.0 μm (Chan et al. 2004). However, it is still unknown whether the association between PM and HRV differs by particle size. To shed light on this question, we used a panel of cardiac and hypertensive patients to study which size fractions had greater effects on HRV reduction among PM with aerodynamic diameters between 0.3 μm and 1.0 μm (PM_0.3–1.0_), between 1.0 μm and 2.5 μm (PM_1.0–2.5_), and between 2.5 μm and 10 μm (PM_2.5–10_).

## Materials and Methods

### Subjects.

This panel study was designed to monitor changes in PM mass concentrations and HRV indices continuously and simultaneously in our study subjects from November 2002 through March 2003. There were 10 patients with CHD and 16 patients with either prehypertension or hypertension in this study. These patients were recruited from the cardiology section, Department of Internal Medicine, National Taiwan University Hospital, and a community health center in the Taipei metropolitan area (Hsin-Chuang Health Center). All the CHD patients had history of angina pectoris and/or acute myocardial infarction and had cardiac catheterized and percutaneous transluminal coronary angioplasty during the year before our panel study. The prehypertensive/hypertensive patients’ hypertension statuses were identified by their annual health checkup at the health center. Each subject’s sex, age, body mass index (BMI), smoking status, and medical history were collected by a face-to-face interviewed questionnaire. Each subject’s current health status was obtained from medical charts and examinations. Professionally trained nurses performed sitting blood pressure measurements for each patient with mercury sphygmomanometer. The criteria of Chobanian et al. (2003) were used to define eight subjects as hypertensive [systolic blood pressure (SBP) ≥140 mmHg or diastolic blood pressure (DBP) ≥90 mmHg] and another eight subjects as prehypertensive (SBP 120–139 mmHg or DBP 80–89 mmHg). To reduce confounding effects in this study, we excluded the following subjects from our recruitment: current smokers; patients with hyperthyroidism, acute cardiopulmonary failure, or paced cardiac rhythm; and patients with current medications of anticholinergics, beta-blockers, or antiar-rhythmic agents. The ethics committee of the National Taiwan University Hospital approved this study. Written informed consent was obtained from each participant before the study embarked.

### Continuous Holter monitoring and tape processing.

We performed continuous ambulatory electrocardiographic (ECG) monitoring for each subject by using a PacerCorder 3-channel device (model 461A; Del Mar Medical Systems LLC, Irvine, CA, USA) with a sampling rate of 250 Hz (4 msec). We sent ECG tapes to National Taiwan University Hospital and used a Delmar Avionics model Strata Scan 563 (Irvine, CA, USA) to do the analysis. The ECG wave complex (QRS) was classified as normal sinus rhythm, arterial or ventricular premature beats, and noise by comparing the adjacent QRS morphologic features. The normal-to-normal (NN) intervals were deduced from the adjacent normal sinus beats. The NN interval time series were then transferred to a personal computer and postprocessed by a program written in Matlab language (version 5.2; MathWorks Inc., Natick, MA, USA). The missing intervals of the raw NN data were linearly interpolated and resampled at 4 Hz by the Ron-Berger method (Berger et al. 1986). Each 5-min segment of NN intervals was taken for HRV analysis. The time domain measurements of HRV were the SD of NN intervals (SDNN) and the square root of the mean of the sum of the squares of differences between adjacent NN intervals (r-MSSD). The frequency-domain measurements of HRV included low frequency (LF; 0.04–0.15 Hz), high frequency (HF; 0.15–0.40 Hz), and LF:HF ratio, which were calculated by Welch’s averaged peri-odogram of the NN intervals (Task Force 1996; Welch 1967). Fast Fourier transformation was performed to estimate power spectral density. To avoid sleep effects on HRV, in our data analysis we used approximately 16-hr Holter measurements when the subjects were awake between 0700 hr and 2300 hr. Each subject provided approximately 192 successful segments of 5-min HRV measurements for further data analysis.

### Personal exposure measurements.

Personal exposures to different sizes of PM were measured persistently by using a personal dust monitor (DUST-check portable dust monitor, model 1.108; Grimm Labortechnik Ltd., Ainring, Germany), which measured and recorded 1-min mass concentrations of PM_0.3–1.0_, PM_2.5_, PM_10_, as well as ambient temperature and humidity. The DUST-check portable dust monitor measured number concentrations by particle’s light-scattering property and used a correction factor to derive mass concentrations from reference aerosols with a density of 0.92 and reflective index of 1.45. Collocated Rupprecht and Patashnick 1400a tapered element oscillating micro-balance (TEOM) samplers (Thermo Electron Corporation, East Greenbush, NY, USA) were used to calibrate the mass concentrations of PM_10_, PM_2.5_, and PM_0.3–1.0_ measured by our DUST-check monitor before and after study. Concurrent PM measurements by the TEOM and the DUST-check monitor showed good association between these two monitors for three size fractions: PM_10_ (*r*^2^ = 0.90), PM_2.5_ (*r*^2^ = 0.91), and PM_0.3–1.0_ (*r*^2^ = 0.79). However, in the concurrent PM measurements by two monitors, the DUST-check monitor reported approximately 10, 15, and 30% more PM_10_, PM_2.5_, and PM_0.3–1.0_ mass concentration, respectively, than did the TEOM monitor.

To measure our patients’ personal PM exposures, a technician carrying a DUST-check monitor was asked to accompany each subject from 0700 hr to 2300 hr. The sampling inlet was kept at a distance of approximately 1–2 m away from each study subject, depending on the subject’s activities. The technician also recorded subjects’ time–activity patterns, such as walking, sitting, sleeping, dining, and environmental tobacco smoke exposures during daytime. After sampling, we obtained mass concentrations of PM_2.5–10_ by subtracting PM_2.5_ concentrations from PM_10_ concentrations recorded in our monitors. We obtained mass concentrations of PM_1.0–2.5_ by subtracting PM_0.3–1.0_ concentrations from PM_2.5_ concentrations recorded in our monitors. By summarizing 1-min PM_2.5–10_, PM_1.0–2.5_, and PM_0.3–1.0_ concentrations to 1-hr moving averages between 0700 hr and 2300 hr, we obtained approximately 1,000 segments of PM concentrations for each subject in our data analysis.

### Statistical analysis.

We first plotted PM by HRV indices for each subject to determine if there were observed associations between these two variables, and if there were any outliers that heavily influenced such associations. We also used stepwise multiple regressions without PM to determine key HRV-related personal covariates with a *p*-value < 0.15. The covariates that changed the estimated effect of PM by > 10% were included in our final models with PM measurements. We then applied linear mixed-effects regression models to examine the association between PM and HRV for cardiac and hypertensive patients separately and jointly by running S-PLUS 2000 (MathSoft Inc., Cambridge, MA, USA). In our data analysis, we treated each subject’s sex, age, BMI, and hour of day as time-invariant variables, whereas PM_2.5–10_, PM_1.0–2.5_, PM_0.3–1.0_, temperature, humidity, and HRV were treated as time-varying variables. The outcome variables were SDNN, r-MSSD, LF, HF, and LF:HF ratio, and the exposure variables were 1- to 4-hr moving averages of PM_2.5–10_, PM_1.0–2.5_, and PM_0.3–1.0_. All HRV indices except LF:HF ratio were log_10_-transformed for further data analysis. In our mixed-effects models, we treated subject’s sex, age, BMI, hour of day, temperature, humidity, and PM as fixed effects and each subject as a random effect. We used smoothing spline in S-PLUS to plot outcome variables against temperature and humidity to determine whether their relation was linear or nonlinear. Linear terms were chosen to control temperature and humidity in our final models because our diagnostic plots showed a linear relation between outcome variables and meteorologic variables. Single-pollutant mixed-effects models were used to determine pollution effects for PM_2.5–10_, PM_1.0–2.5_, and PM_0.3–1.0_ separately. Multipollutant mixed-effects models were used to determine what size fractions had greater pollution effects among PM_2.5–10_, PM_1.0–2.5_, and PM_0.3–1.0_. The first-order auto-regressive model (AR1) was chosen to adjust temporal autocorrelation of HRV measurements because residuals plots showed that AR1 was sufficient to remove the autocorrelation of the observed outcome series. Model selections were based on the criteria of minimizing Akaike’s information criterion (Akaike 1974). Pollution effects are expressed as percent changes in HRV by interquartile changes in PM concentrations.

## Results

As shown in Table 1, the ages of our 26 study subjects were 61–72 years among 10 cardiac patients and 52–76 years among 16 prehypertensive/hypertensive patients (the hypertensive group). Their mean BMIs were 25.6 kg/m^2^ for the cardiac patients and 24.4 kg/m^2^ for the hypertensive group. Our study subjects’ HRV indices, PM exposures, and meteorologic conditions during the study period are summarized in Table 2. The cardiac patients had significantly higher values of HRV indices than did the hypertensive group. Moreover, the cardiac patients had significantly higher PM_2.5–10_ exposures but lower PM_1.0–2.5_ and PM_0.3–1.0_ exposures than did the hypertensive group. On average, PM_0.3–1.0_ levels of 26.8 μg/m^3^ in the cardiac patients and 37.2 μg/m^3^ in the hypertensive group accounted for 49.5 and 58.3% of PM_10_ mass concentrations in their respective groups. The interquartile ranges of PM_0.3–1.0_ exposures spanned 28.3 μg/m^3^ for the cardiac patients and 27.2 μg/m^3^ for the hypertensive group. Pearson correlations between any two combinations of PM_2.5–10_, PM_1.0–2.5_, and PM_0.3–1.0_ showed moderate correlations between PM_0.3–1.0_ and PM_1.0–2.5_ (*r* = 0.65) and between PM_1.0–2.5_ and PM_2.5–10_ (*r* = 0.51) only. Hourly temperature varied from 17.6°C to 33.0°C, and hourly relative humidity varied from 28.6 to 80.5% during the study period.

The associations between PM and time-domain HRV indices estimated by mixed-effects models are listed in Table 3. With sex, age, BMI, hour of day, temperature, and humidity being adjusted in our mixed-effects models, PM_0.3–1.0_ exposures significantly decreased SDNN and r-MSSD for both the cardiac patients and the hypertensive group. By contrast, PM_2.5–10_ and PM_1.0–2.5_ exposures were not associated with SDNN or r-MSSD in our study subjects. For cardiac patients, interquartile increases in PM_0.3–1.0_ with 2- to 4-hr moving-average exposure were associated with 2.87–4.88% decreases in SDNN. Their r-MSSDs were decreased by 4.43–8.25% with 1- to 4-hr moving averages, respectively. For the hypertensive group, interquartile increases in PM_0.3–1.0_ with 1- to 4-hr moving averages exposure accounted for about 1.49–1.79% decreases in SDNN and 2.73–5.07% decreases in r-MSSD. The greatest decreases in time-domain HRV indices occurred with 3-hr moving averages for the cardiac patients and 4-hr moving averages for the hypertensive group. We examined the time course of PM exposures only up to 4-hr moving averages because available data became substantially decreased for calculating moving averages > 5 hr.

The associations between PM and frequency-domain HRV indices by our mixed-effects models are list in Table 4. For the cardiac patients, interquartile increases in PM_0.3–1.0_ exposures significantly decreased LF by 3.83% with 3-hr moving averages and HF by 5.28% with 2-hr moving averages. For the hypertensive group, interquartile increases in PM_0.3–1.0_ exposures decreased LF by 2.32% and 1.86% with 1-hr and 2-hr moving averages, respectively. Their respective HF values decreased by 2.84 and 3.29% by interquartile increases in PM_0.3–1.0_ exposures with 1- to 3-hr moving averages. By contrast, PM_2.5–10_ and PM_1.0–2.5_ exposures were not associated with LF or HF in our study subjects. No association was observed between PM of all three size ranges and the LF:HF ratios in our study subjects.

**Figure 1:**
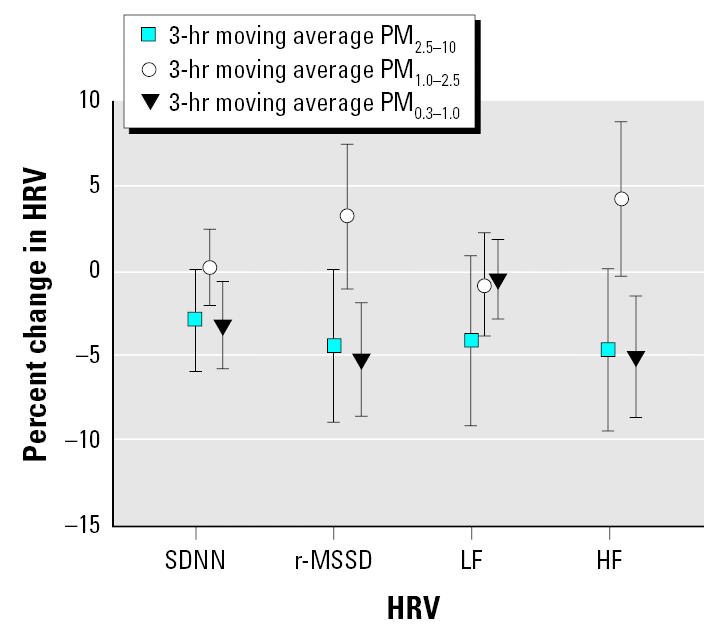
Estimated percent changes in HRV by interquartile increase in PM_0.3–1.0_, PM_1.0–2.5_, and PM_2.5–10_ exposures at 3-hr moving averages for 26 study subjects using multipollutant mixed-effects models. Error bars indicate 95% confidence intervals.

Because our study subjects are exposed to PM_10_, PM_2.5_, and PM_0.3–1.0_ simultaneously during the panel study, their exposures to three size fractions of PM can be treated as copollutants in our multipollutant models. We found that PM_0.3–1.0_ effects on HRV reduction in multipollutant models remained as significant as those in the single-pollutant models. By contrast, both PM_2.5–10_ and PM_1.0–2.5_ were not associated with HRV reduction in the multipollutant models. Figure 1 lists one exemplary result of our multipollutant models, which shows the percent reduction in HRV by PM_2.5–10_, PM_1.0–2.5_, and PM_0.3–1.0_ using 3-hr moving averages of these three PM fractions and all 26 subjects in this study. As shown in Figure 1, our subjects’ SDNN, r-MSSD, and HF values were decreased by about 3.16, 5.20, and 5.05% for interquartile increases in 3-hr PM_0.3–1.0_ moving averages, respectively. To further determine whether disease status could modify the association between PM and HRV, we combined the data of cardiac and hypertensive patients together and put them into our multi-pollutant models with and without the disease status as a variable in the models. We found that the addition of disease status did not significantly change the coefficients of PM in our multipollutant models (data not shown).

## Discussion

This is the first study to report that PM_0.3–1.0_ measured in mass concentrations had effects on reducing HRV among cardiac, prehypertensive, and hypertensive patients. This study supports that PM_0.3–1.0_ had effects on decreasing HRV indices in susceptible populations, as we reported in a previous panel study using number concentrations of submicrometer particles with a size range of 0.02–1.0 μm (Chan et al. 2004). One toxicologic study also reported that PM_1.0_ induced more production of interleukin-8, lipid peroxidation, and tumor necrosis factor-α in mouse macrophage RAW 264.7 cells than did PM_2.5–10_ or PM_1.0–2.5_ (Huang et al. 2003).

The time courses of PM_0.3–1.0_ on HRV in cardiac and hypertensive patients ranging from 1 to 4 hr are in agreement with the findings of previous studies (Chan et al. 2004; Gold et al. 2000; Magari et al. 2001, 2002). These results indicate that PM_0.3–1.0_ can have acute effects on cardiac autonomic function. It has been reported that particles can affect both sympathetic and parasympathetic nervous systems directly immediately after exposures (Kodavanti et al. 2000; Lai and Kou 1998). One possible pathway of such a mechanism is the rapid passage of inhaled particles with diameters < 100 nm into the blood circulation (Nemmar et al. 2001, 2002). Under appropriate circumstances, the activation of pulmonary neural reflexes secondary to PM interactions in autonomic tone may contribute to the instability of vascular plaque or initiate cardiac arrhythmias. Such a direct effect of PM represents a plausible explanation for the occurrence of rapid cardiovascular responses in 1-hr moving average of PM_0.3–1.0_ exposure. Another possible pathophysiologic link between PM and less acute effects of cardiovascular responses is that inhaled particles may exacerbate the autonomic function of the heart via induced inflammation in lung and proinflammatory cytokine expression in cardiac macrophages (Stone and Godleski 1999). Previous studies also reported that ultrafine particles deposited in the alveoli might increase blood coagulation via mechanisms of pulmonary inflammation or direct action on red blood cells (Donaldson et al. 2001; Peters et al. 1997). This subsequently may contribute to a systemic inflammatory state, which may in turn be capable of activating hemostatic pathways, impairing vascular function, and causing atherosclerosis. Accordingly, we believe particle-induced pulmonary inflammation can also indirectly result in HRV changes or autonomic imbalance in the delayed phase after PM_0.3–1.0_ exposures. This may explain why HRV decrease reached its peak at 3–4 hr after PM_0.3–1.0_ exposure in our study.

There is a growing recognition that autonomic dysfunction plays an important role in cardiovascular mortality. Autonomic nervous system changes in HRV may increase the likelihood of sudden cardiac death (Task Force 1996). Decrease in HRV is also a strong predictor of cardiac mortality (La Rovere et al. 2003). Because the cardiac autonomic alteration included both time-domain and frequency-domain HRV indices in this study, we believe that cardiovascular diseases may be increased by PM_0.3–1.0_-induced decreases in autonomic nervous system control or the withdrawal of vagal activity (Bigger et al. 1992; Kleiger et al. 1987). However, it was still unclear whether short-term and small HRV fluctuations caused by PM_0.3–1.0_ exposures will eventually lead to cardiac deaths. Because cardiac death is a consequence of a complex interaction between the autonomic nervous system, a myocardial substrate altered in the course of disease processes, and myocardial vulnerability leading to arrhythmogenic or ischemic response, the presence of a single cardiac alteration is usually not sufficient to trigger cardiac death (Zareba et al. 2001). Further studies on environmental cardiology are needed to determine whether the PM_0.3–1.0_-associated HRV fluctuations observed in panel studies have meaningful implications of cardiovascular mortality clinically.

The following limitations of our study design must be considered in explaining our findings of PM_0.3–1.0_ effects on reducing HRV in this study. First, the lack of information on personal exposure to other air pollutants, such as nitrogen dioxide, carbon monoxide, ozone, and sulfur dioxide may confound the observed associations between PM_0.3–1.0_ and HRV indices. Because these air pollutants are usually correlated with PM, they can bias our study outcomes toward either positive or null results (Zeger et al. 2000; Zeka and Schwartz 2004). Therefore, we cannot entirely rule out the effects of these air pollutants on reducing HRV in this study. Second, the observed PM_0.3–1.0_ effects on HRV reduction may be due to differences in particle components rather than particle sizes. The lack of measuring chemical and biologic components in our subjects’ PM exposures prevents us from differentiating particle size from particle components in HRV reduction in our study. Third, it is possible that the DUST-check monitor may have been turned off in high PM environments, such as busy traffic zones, during the monitoring period. More frequent calibrations of the DUST-check monitor during the study could have been more temporally supportive to validate continued accuracy although a comparison with a collocated TEOM sample was made to calibrate DUST-check monitors before and after the study. Fourth, we cannot exclude the confounding effects of respiration on the association between PM_0.3–1.0_ and HRV because our subjects’ breathing patterns were not measured in our study and the quantity, periodicity, and timing of vagal cardiac outflow are associated with variations of respiratory depth and interval (Yasuma and Hayano 2004). Fifth, the technician’s presence may also alter the subjects’ psychology and autonomic system, and then alter their behaviors, including breathing patterns and heart rates. Sixth, the use of 5-min segments of NN intervals eliminates the opportunity to evaluate HRV frequencies > 5 min and to compare our results against those findings using different averaging times, such as 24-hr SDNN and standard deviation of the averages of NN intervals in all 5-min segments of the entire recording. Therefore, our results did not preclude the findings of previous daily time-series studies on respiratory and cardiovascular mortality, which have generally observed exposure lag structures to be 1–5 or more days, because this study examined time course only within 1 day.

Regardless of these limitations, we believe our data generally support the conclusion that PM_0.3–1.0_ is an environmental stressor, which may contribute to the fluctuations of HRV indices and trigger a cascade of events by increasing autonomic function imbalance, and may potentially lead to ischemia or fatal arrhythmia in patients with underlying CHD, prehypertension, or hypertension. Cardiac patients together with hypertensive adults are susceptible to PM_0.3–1.0_ and should be considered a high-risk target population in planning future public health abatement measures against PM_0.3–1.0_ pollution.

## Figures and Tables

**Table 1:** Basic characteristics of 26 study subjects.

Characteristic	Cardiac patients	Hypertensive patients
Sex (*n*)		
Female	1	11
Male	9	5
Age (years)	68.1 ± 3.6 (61–72)	68.8 ± 6.6 (52–76)
BMI (kg/m^2^)	25.6 ± 4.8 (19.5–34.7)	24.4 ± 2.8 (20.6–31.8)
Heart rate (beats/min)	79.6 ± 14.8 (48.7–123.0)	77.4 ± 11.9 (47.9–114.8)
Health status (*n*)		
CHD	10	0
Prehypertension^a^	0	8
Hypertension^b^	0	8

Values are mean ± SD (range) unless otherwise noted.

a Prehypertension: SBP 120–139 mmHg or DBP 80–89 mmHg.

b Hypertension: SBP ≥ 140 mmHg or DBP ≥90 mmHg.

**Table 2:** Summary statistics for HRV indices, air pollution levels, and meteorologic variables (mean ± SD).

Variable	Cardiac patients	Hypertensive patients	*p*-Value^a^
Time-domain HRV			
Log_10_ SDNN (msec)	1.53 ± 0.24	1.56 ± 0.21	< 0.0001
Range	0.71–2.01	0.73–2.10	
No.	1,527	2,864	
Log_10_ r-MSSD (msec)	0.97 ± 0.29	1.00 ± 0.27	0.0002
Range	0.39–1.83	0.40–1.86	
No.	1,527	2,864	
Frequency-domain HRV			
Log_10_ LF (msec^2^)	2.15 ± 0.57	2.21 ± 0.49	0.0006
Range	0.05–3.91	0.38–4.16	
No.	1,527	2,864	
Log_10_ HF (msec^2^)	1.97 ± 0.65	2.07 ± 0.63	< 0.0001
Range	0.44–3.88	0.33–4.03	
No.	1,527	2,864	
LF:HF ratio	2.75 ± 3.33	2.14 ± 2.14	< 0.0001
Range	0.04–40.12	0.06–17.81	
No.	1,527	2,864	
Air pollutants			
PM_2.5–10_ 1-hr mean (μg/m^3^)	16.4 ± 10.7	14.0 ± 11.1	< 0.0001
Interquartile range	14.8	11.9	
Range	0.7–59.6	0.3–66.5	
No.	1,084	2,273	
PM_1.0–2.5_ 1-hr mean (μg/m^3^)	10.9 ± 8.5	12.6 ± 7.8	< 0.0001
Interquartile range	10.8	7.9	
Range	0.9–48.8	0.5–62.8	
No.	1,084	2,273	
PM_0.3–1.0_ 1-hr mean (μg/m^3^)	26.8 ± 25.9	37.2 ± 25.8	< 0.001
Interquartile range	28.3	27.2	
Range	1.4–136.2	1.3–196.4	
No.	1,084	2,273	
Meteorologic variables			
Temperature (°C)	25.0 ± 3.5	26.3 ± 3.6	< 0.0001
Range	18.4–31.4	17.6–33.0	
No.	1,248	2,568	
Relative humidity (%)	55.4 ± 8.5	57.0 ± 8.2	< 0.0001
Range	28.6–74.2	39.5–80.5	
No.	1,248	2,568	

a Difference between cardiac and hypertensive patients was tested by *t*-test.

**Table 3:** Percent changes (95% confidence interval)^a^ in time-domain HRV for interquartile increase in PM exposures estimated by mixed-effects models.

	Cardiac patients			Hypertensive patients		
Exposure matrix	PM_2.5–10_	PM_1.0–2.5_	PM_0.3–1.0_	PM_2.5–10_	PM_1.0–2.5_	PM_0.3–1.0_
SDNN						
1-hr moving	−1.73 (−3.53 to 0.08)	−1.36 (−3.56 to 0.85)	−1.50 (−3.45 to 0.45)	−2.64 (−3.93 to 0.55)	−2.39 (−5.40 to 0.62)	−1.63* (−2.42 to −0.85)
2-hr moving	−1.97 (−4.43 to 0.49)	−2.40 (−5.13 to 0.32)	−2.87* (−5.23 to −0.51)	−3.51 (−7.87 to 0.85)	−2.47 (−5.19 to 0.26)	−1.75* (−2.74 to −0.76)
3-hr moving	−1.70 (−4.39 to 0.98)	−4.00 (−8.11 to 0.10)	−4.88* (−7.79 to −1.97)	−2.74 (−6.22 to 0.74)	−1.83 (−5.17 to 1.52)	−1.49* (−2.62 to −0.36)
4-hr moving	−1.75 (−5.42 to 1.92)	−4.50 (−9.52 to 0.52)	−3.95* (−7.59 to −0.31)	−2.49 (−6.13 to 1.15)	−2.36 (−5.81 to 1.10)	−1.79* (−2.97 to −0.61)
r-MSSD						
1-hr moving	−4.39 (−9.54 to 0.03)	−4.39 (−8.89 to 0.10)	−4.43* (−8.10 to −0.77)	−2.53 (−5.10 to 0.04)	−3.12 (−7.27 to 1.04)	−2.73* (−4.39 to −1.08)
2-hr moving	−4.36 (−8.99 to 0.27)	−5.68 (−11.83 to 0.46)	−6.91* (−11.41 to −2.40)	−5.42 (−10.92 to 0.09)	−4.33 (−9.91 to 1.24)	−3.37* (−5.44 to −1.30)
3-hr moving	−4.20 (−9.02 to 0.61)	−6.30 (−12.73 to 0.14)	−8.25* (−13.64 to −2.87)	−3.15 (−6.32 to 0.03)	−2.59 (−5.37 to 0.18)	−3.36* (−5.65 to −1.07)
4-hr moving	−2.70 (−9.24 to 3.84)	−3.99 (−13.07 to 5.10)	−4.94 (−11.60 to 1.72)	−4.23 (−8.88, to 0.42)	−5.17 (−10.79 to 0.44)	−5.07* (−7.55 to −2.59)

a Coefficients are expressed as percent changes for interquartile changes in PM exposures in models adjusting for sex, age, BMI, hour of day, temperature, and humidity.

* *p* < 0.05.

**Table 4:** Percent changes (95% confidence interval)^a^ in frequency-domain HRV for interquartile increase in PM exposures estimated by mixed-effects models.

	Cardiac patients			Hypertensive patients		
Exposure matrix	PM_2.5–10_	PM_1.0–2.5_	PM_0.3–1.0_	PM_2.5–10_	PM_1.0–2.5_	PM_0.3–1.0_
LF						
1-hr moving	−1.85 (−4.33 to 0.62)	−1.65 (−4.67 to 1.37)	−1.91 (−4.51 to 0.69)	−4.38 (−8.78 to 0.03)	−3.72 (−7.84 to 0.30)	−2.32* (−3.58 to −1.07)
2-hr moving	−3.87 (−8.22 to 0.47)	−3.10 (−6.84 to 0.64)	−2.39 (−5.57 to 0.79)	−5.23 (−10.95 to 0.05)	−3.23 (−6.71 to 0.26)	−1.86* (−3.46 to −0.25)
3-hr moving	−2.98 (−6.65 to 0.69)	−4.10 (−9.00 to 0.79)	−3.83* (−8.29 to −0.36)	−3.34 (−1.72 to 0.04)	−1.75 (−3.87 to 0.37)	−1.11 (−2.89 to 0.66)
4-hr moving	−3.11 (−8.22 to 1.99)	−4.96 (−11.97 to 2.06)	−2.82 (−7.76 to 2.12)	−2.96 (−6.63 to 0.71)	−2.61 (−5.26 to 0.04)	−1.53 (−3.43 to 0.37)
HF						
1-hr moving	−4.46 (−9.23 to 0.32)	−3.66 (−8.25 to 0.93)	−3.94 (−8.00 to 0.12)	−4.92 (−9.94 to 0.10)	−3.97 (−8.37 to 0.43)	−3.10* (−4.95 to −1.25)
2-hr moving	−4.41 (−9.55 to 0.72)	−4.86 (−10.52 to 0.81)	−5.28* (−10.20 to −0.36)	−6.07 (−12.28 to 0.13)	−4.28 (−9.15 to 0.60)	−3.29* (−5.61 to −0.96)
3-hr moving	−3.80 (−9.12 to 1.53)	−3.31 (−10.36 to 3.74)	−4.30 (−10.18 to 1.57)	−1.94 (−5.44 to 1.55)	−1.54 (−4.63 to 1.56)	−2.84* (−5.41 to −0.26)
4-hr moving	−3.39 (−10.62 to 3.84)	−2.15 (−12.03 to 7.73)	−2.38 (−9.49 to 4.74)	−2.78 (−6.78 to 1.21)	−3.55 (−9.04 to 1.94)	−3.91 (−8.72 to 0.89)
LF:HF ratio						
1-hr moving	8.45 (−3.48 to 20.38)	3.71 (−14.09 to 21.52)	5.75 (−4.06 to 15.56)	5.94 (−3.27 to 15.15)	3.43 (−8.77 to 15.63)	7.54 (−2.45 to 17.54)
2-hr moving	1.66 (−15.22 to 18.55)	−6.84 (−29.89 to 16.21)	4.93 (−8.03 to 17.89)	10.70 (−2.19 to 23.59)	7.55 (−6.34 to 21.44)	10.16 (−1.28 to 21.59)
3-hr moving	11.69 (−7.27 to 30.64)	−24.06 (−56.35 to 8.24)	−9.11 (−27.76 to 9.55)	−1.51 (−17.02 to 14.00)	−3.32 (−21.22 to 14.57)	14.49 (−1.80 to 30.77)
4-hr moving	8.18 (−17.22 to 33.57)	−47.72 (−96.30 to 1.17)	−10.38 (−34.89 to 14.12)	3.41 (−16.91 to 23.74)	4.32 (−18.64 to 27.29)	16.58 (−0.75 to 33.91)

a Coefficients are expressed as percent changes for interquartile changes in PM exposures in models adjusting for sex, age, BMI, hour of day, temperature, and humidity.

* *p* < 0.05.
